# Haptoglobin Genotype and Risk Markers of Cardiovascular Disease in Patients with Chronic Kidney Disease

**DOI:** 10.1155/2013/650847

**Published:** 2013-03-16

**Authors:** Charlotte Strandhave, My Svensson, Henrik Krarup, Jeppe Hagstrup Christensen

**Affiliations:** ^1^Department of Nephrology, Aalborg Hospital, Aarhus University Hospital, Moelleparkvej 4, 9000 Aalborg, Denmark; ^2^Center for Cardiovascular Research, Aalborg Hospital, Aarhus University Hospital, Aalborg, Denmark; ^3^Department of Nephrology, Aarhus University Hospital, 8200 Aarhus N, Denmark; ^4^Department of Clinical Biochemistry, Aalborg Hospital, Aarhus University Hospital, Aalborg, Denmark

## Abstract

Sudden cardiac death and atherosclerosis have a major impact on cardiovascular mortality in chronic kidney disease (CKD). Inflammation with elevated high-sensitive C-reactive protein (hs-CRP) is involved in both sudden cardiac death and atherosclerosis, and decreased heart rate variability (HRV) is a predictor of both sudden cardiac death and atherosclerosis. Haptoglobin (Hp) is characterised by three genotypes (1-1, 2-1, and 2-2) with different antioxidant abilities. The aim was to examine whether HRV and hs-CRP were associated with Hp genotype in CKD patients. Fifty-six patients with CKD stage 2–5 were included. Hp genotype was determined by high-performance liquid chromatography. HRV was analysed from the 24 h Holter recordings. Hs-CRP was measured using an immunoturbidimetric assay. The results show that the HRV indices SDNN and SDANN were significantly lower in the Hp 2-2 patients (*P* = 0.02 and 0.04, resp.). In an adjusted linear regression model, Hp 2-2 was associated with both SDNN (*P* = 0.005) and SDANN (*P* = 0.01). Hs-CRP was higher in the Hp 2-2 patients (*P* = 0.002). In an adjusted linear regression model, the association between Hp 2-2 and hs-CRP remained significant (*P* = 0.003). In conclusion, a negative association was observed between Hp 2-2 and HRV, and Hp 2-2 was positively associated with hs-CRP in CKD patients.

## 1. Introduction

Cardiovascular disease (CVD) including sudden cardiac death (SCD) [1] is a major cause of mortality in chronic kidney disease (CKD) [2]. Apart from left ventricular dysfunction [1], unique factors such as autonomic imbalance and inflammation contribute to the high incidence of SCD in CKD [3]. Traditional risk factors for atherosclerosis [4], such as hypertension and dyslipidaemia, can to some extent explain the increased risk of atherosclerosis in CKD [5]. However, nontraditional risk factors like oxidative stress and inflammation may also be of importance [6].

 Heart rate variability (HRV) is a reliable, noninvasive measurement of autonomic tone of the heart [7]. A reduction in HRV reflects an autonomic imbalance towards decreased vagal tone and subsequent sympathetic overactivity [3]. Decreased HRV is a predictor of SCD in patients with CKD [8]. Furthermore, attenuated HRV is associated with atherosclerosis in CKD [9].

 Chronic, low-grade inflammation is involved in the development of both SCD [10] and atherosclerosis [11]. The most extensively examined marker of inflammation is high-sensitive C-reactive protein (hs-CRP), which is a predictor of cardiovascular mortality in different populations [12, 13] including patients with CKD [14]. Hs-CRP may even be a risk marker for SCD [10, 15].

 Haptoglobin (Hp) is an abundant acute-phase protein as well as an innate antioxidant involved in the scavenging of free haemoglobin [16]. Thus, Hp prevents iron-mediated formation of free reactive oxygen species known to initiate oxidative stress [17]. In humans, Hp is characterised by molecular heterogeneity with three common genotypes: 1-1, 2-2, and 2-1 [18]. The antioxidant ability of Hp is genotype dependent with Hp 2-2 being the weakest antioxidant [19]. There is evidence establishing Hp 2-2 as a predictor of CVD in diabetes mellitus [20, 21]. However, results are inconsistent regarding Hp genotypes and cardiovascular mortality in patients with CKD [22, 23]. A study in patients with ischemic heart disease (IHD) revealed a positive association between low HRV and Hp 2-2 [24].

 The aim of the present study was to determine whether Hp genotype was associated with the cardiovascular risk markers HRV and hs-CRP in patients with CKD.

## 2. Material and Methods

### 2.1. Study Population

Sixty-four patients from the Outpatient Clinic at the Department of Nephrology, Aalborg Hospital, Denmark, were included. Inclusion criterion was CKD stage 2–5. Exclusion criteria were the start of dialysis within three months, nephrotic syndrome, malignancy, uncontrolled hypertension, or a previous renal transplantation. Patients were also excluded if they had an ongoing infection, a myocardial infarction during the last six months, or supraventricular arrhythmia.

 Six patients with diabetes were excluded. Two patients were excluded due to technical problems. Thus, the final analysis comprised 56 patients.

 The causes of CKD were chronic glomerulonephritis (32%), hypertensive nephropathy (23%), polycystic kidney disease (13%), and other causes including interstitial nephritis and chronic pyelonephritis (32%).

 The study was conducted in accordance with Danish Legislation and the Declaration of Helsinki and approved by Research Ethics Committee of The North Denmark Region. Signed informed consent was obtained from all participants.

### 2.2. Laboratory Measurements

Blood samples were drawn after an overnight fast. All routine analyses were performed according to the standard procedures in the hospital laboratory. Creatinine clearance was calculated using a 24 h urine collection and a blood sample for serum creatinine. Hs-CRP was measured using an ADVIA 1650 analyzer (Bayer Corp., Pittsburgh, PA, USA) and an immunoturbidimetric assay (Randox Laboratories Ltd., Antrim, UK). Detection level was 0.2 mg/L. The analysis was conducted in accordance with the instruction of the manufacturer. 

 The Hp genotype was determined by high-performance liquid chromatography as previously described [25], using a Superdex 200 HR 10/30 column for chromatographic separation (Amersham Pharmacia Biotech AB, Birkerød, Denmark). Phosphate-buffered saline (50 mmol/L sodium phosphate, 150 mmol/L sodium chloride, and 0.01% sodium azide; pH 7.3) was used as the mobile phase. Flow rate was 1 mL/min (76.4 cm/hour). Ten *μ*L was used for analysis. Hp genotype was determined from the curve shape and relative retention time of the chromatograms.

### 2.3. Heart Rate Variability Measurement

A 24 h Holter recording was obtained on a flash card using a 3-channel digital monitor DL-700 (Diagnostic Monitoring, Santa Ana, CA, USA), and time domain variables were analysed using commercially available software (Diagnostic Monitoring, Santa Ana, CA, USA). If more than 20% of QRS complexes were abnormal or of poor technical quality, the analysis was excluded in the overall HRV analysis. The time domain variables were identified as follows: (1) mean of all normal-to-normal RR intervals, (2) standard deviation (SD) of all normal-to-normal RR intervals (SDNN), (3) mean of the SDs of all normal-to-normal RR intervals (SDNNindex), (4) SD of the mean normal-to-normal RR intervals for each 5-minute period (SDANN), (5) percentage of successive RR interval differences >50 ms (pNN50), and (6) square root of the mean of the sum of squares of differences between adjacent RR intervals (RMSSD). Heart rate was calculated as the inverse value of the RR variable.

### 2.4. Blood Pressure Measurement

Blood pressure was obtained with a 24 h ambulatory BP device, TM 2412 (A&D Co. Ltd., Tokyo, Japan). For analysis, average values during daytime for systolic and diastolic blood pressure were calculated.

### 2.5. Statistical Analysis

The statistical analysis was performed using Stata software, version 10 (StataCorp LP, TX, USA). Continuous data were expressed as mean values ± SD if normal distributed. Nonnormal distributed data were log transformed and expressed as geometric mean and ranges. The Hp alleles were tested for Hardy-Weinberg equilibrium using an asymptotic Hardy-Weinberg equilibrium test.

 For the comparison of SDNN, SDANN, and hs-CRP, respectively, between the three common Hp genotypes, crude one-way analysis of variance was performed.

 Data was divided into two groups according to the presence of the Hp allele 1: one group including Hp 1-1 and Hp 2-1 patients and the remaining group consisting of Hp 2-2 patients. For comparisons of groups, Student's *t*-test was used for continuous variables and Fisher's exact test for categorical variables. Linear regression was used to analyse the effect of Hp 2-2 compared to Hp 1-1 and Hp 2-1 on SDNN, SDANN, and hs-CRP, respectively. The analyses were performed with the Hp group as the independent variable and SDNN, SDANN, and hs-CRP, respectively, as the dependent variables. Adjustments for potential confounders described in previous studies (age, gender, heart rate, beta-blocker treatment, and creatinine clearance for HRV indices and age, gender, body mass index, and smoking status for hs-CRP) [26–28] were performed. Regression coefficients represented a difference between the two Hp genotype groups. A *P* value < 0.05 was considered statistically significant.

## 3. Results

The frequencies of the three common genotypes were Hp 1-1 21%, Hp 2-1 50%, and Hp 2-2 29%, and the Hp allele frequencies (Hp allele 1 0.457 and Hp allele 2 0.544) were in Hardy-Weinberg equilibrium (*χ* ^2^ = 2.07 and *P* = 0.1). The genotype frequencies in the cohort were similar to those reported previously in northwestern European countries (*χ* ^2^ = 3.42 and *P* = 0.2) [29] and similar to those reported previously in a Danish population (*χ* ^2^ = 3.40 and *P* = 0.2) [30].

**Figure 1:**
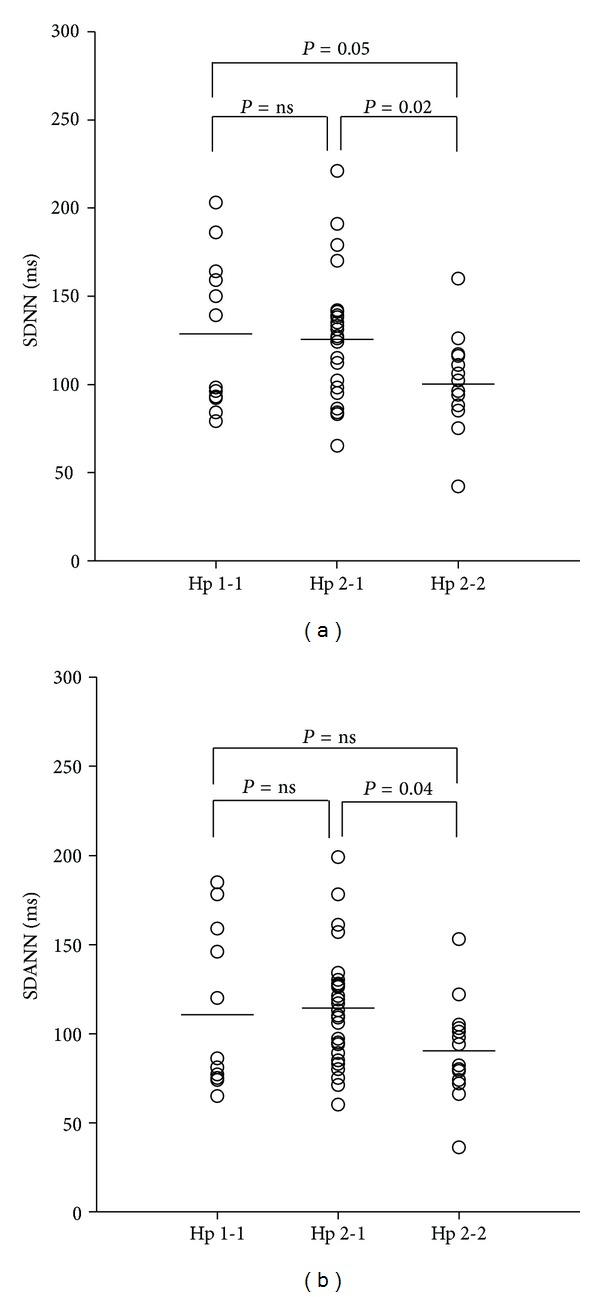
Dot plots showing (a) SDNN (ms) and (b) SDANN (ms) in haptoglobin genotypes 1-1, 2-1, and 2-2, respectively. The lines represent mean values. Abbreviations Hp: haptoglobin.

**Figure 2:**
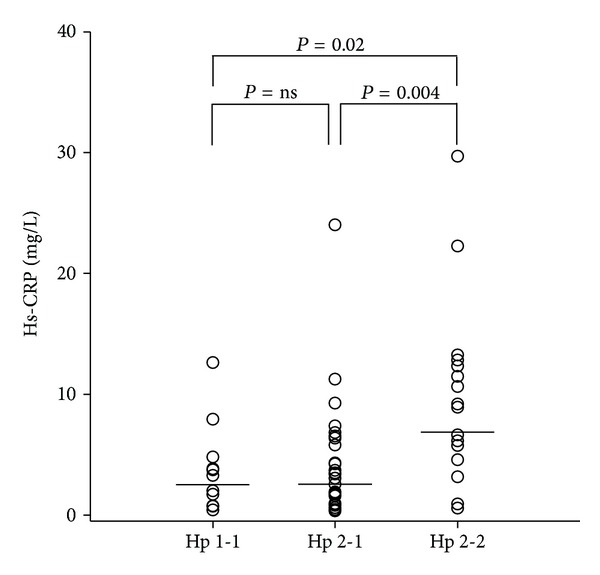
Dot plot showing high-sensitive C-reactive protein (mg/L) in haptoglobin genotypes 1-1, 2-1, and 2-2, respectively. The lines represent mean values. Abbreviations: Hp: haptoglobin and hs-CRP: high-sensitive C-reactive protein.

Figure 1 depicts mean SDNN and SDANN, and Figure 2 mean hs-CRP in the three common Hp genotypes. No differences were demonstrated between Hp 1-1 and Hp 2-1 regarding SDNN, SDANN, and hs-CRP, respectively. However, significant differences were observed between Hp 1-1 and Hp 2-2 and between Hp 2-1 and Hp 2-2 for both SDNN and hs-CRP. However, for SDANN, a significant difference was demonstrated only between Hp 2-1 and Hp 2-2.

Table 1 shows clinical characteristics in the Hp genotypes divided into two groups according to the presence of Hp allele 1. The two groups were generally comparable, although there were fewer women in the Hp 2-2 group.

 SDNN and SDANN were significantly lower in the Hp 2-2 patients compared to the Hp 1-1 and Hp 2-1 patients (Table 1). The other time domain indices RR, SDNNindex, pNN50, and RMSSD showed a similar trend although not statistically significant. Multivariate linear regression analyses of Hp 2-2 and SDNN and SDANN, respectively, were performed adjusting for previously described confounders [26]. Hp 2-2 was negatively and independently associated with both SDNN and SDANN (Table 2). Apart from Hp 2-2, the strongest determinants of SDNN in the model were age (*P* < 0.001) and heart rate (*P* = 0.04), and the strongest determinants of SDANN were age (*P* < 0.001), heart rate (*P* = 0.05), and beta-blocker treatment (*P* = 0.04). After adjusting for age, gender, heart rate, creatinine clearance, and beta-blocker treatment, Hp 2-2 was still significantly associated with both SDNN and SDANN.

 Levels of hs-CRP were significantly higher in the Hp 2-2 patients compared to the Hp 1-1 and Hp 2-1 patients (Table 1). In the multiple linear regression analysis of hs-CRP and Hp 2-2 (Table 2), adjustments were made for confounders known from previous studies [27, 28]. The association between Hp genotype and hs-CRP remained significant although attenuated. Only Hp 2-2 revealed a positive and significant association with hs-CRP in the model.

## 4. Discussion

The present study demonstrated a close association between Hp genotype and the cardiovascular risk markers HRV and hs-CRP in patients with CKD. Thus, patients with Hp 2-2 had significantly lower HRV and higher levels of hs-CRP.

### 4.1. Haptoglobin Genotype and Risk of Sudden Cardiac Death

Kanbay et al. establishes an SCD as one of the most single important causes of death in patients with CKD [3]. Well-described risk factors of SCD in the general population also apply to patients with CKD [3]. However, several unique factors such as autonomic imbalance and inflammation are recognized [15]. Schwartz has established the importance of cardiac autonomic control in the pathogenesis of SCD [31]. HRV is being increasingly used as a valid and easily obtainable prognostic marker of autonomic alterations [7]. As reviewed by Ranpuria et al., HRV is a promising surrogate marker for SCD in patients with CKD and a potential modifiable outcome measure [32].

 In haemodialysis patients, Oikawa et al. found decreased SDNN to be a predictor of all-cause and cardiovascular mortality [8]. Recently, Chandra et al. studied HRV in patients with CKD stage 3–5 demonstrating that lower HRV was independently associated with higher risk of CVD and mortality [33]. Christensen et al. has previously addressed the issue of a possible association between HRV and Hp genotype [24]. In patients with IHD, a significant association between time domain variable RMSSD and Hp 2-2 was shown. RMSSD primarily reflects the short-term vagal modulation of the sinoatrial node. The present study did not show an association between RMSSD and Hp 2-2. However, a highly significant association between Hp 2-2 and SDNN and SDANN, primarily reflecting circadian rhythm and long-term fluctuations, was demonstrated. 

 A relationship between the autonomic nervous system and inflammation has been established showing increase in vagal tone as a counter-response to cytokine synthesis [34]. Tracey [35] first described this cholinergic anti-inflammatory pathway as a plausible, molecular mechanism for the association between HRV and hs-CRP. In a cohort of patients with CKD, Psychari et al. demonstrated a negative association between inflammatory marker interleukin-6 and SDNN and SDANN, respectively [36]. Furthermore, in a large cohort of end-stage renal disease patients, Parekh et al. [15] found a positive association between hs-CRP and SCD, independently of other known cardiovascular risk factors. Direct effects on the vagal conduction system and resulting aggravation of sympathetic tone may contribute to the increased risk of SCD [15]. Being the weaker antioxidant of the three common Hp genotypes, Hp 2-2 may be unable to hinder oxidative damage to the autonomic nervous system, especially in patients with CKD exposed to a prooxidative milieu due to uremic toxins. Thereby, Hp 2-2 may potentially contribute to a decreased HRV.

### 4.2. Haptoglobin Genotype and Risk of Atherosclerosis

Studies have established Hp 2-2 as a potential risk predictor of CVD in patients without CKD. Chapelle et al. [37] showed that more extensive myocardial infarction occurred in Hp 2-2 patients. Delanghe et al. demonstrated that Hp 2-2 patient undergoing coronary bypass surgery needed more grafts and had shorter graft survival time [38]. On the contrary, in a nested control study de Bacquer et al. showed that Hp 1-1 patients had a higher risk of IHD compared to Hp 2-2 and Hp 2-1 patients [39]. Studies on CVD and Hp genotype in diabetes show more consistent results, both in human studies [21] and recently in a mouse model [40]. Levy et al. demonstrated in the Strong Heart study [20] that Hp 2-2 is a major predictor of CVD in patients with diabetes. Yet, in a cross-sectional study of the Framingham Offspring Cohort [41], Levy et al. found that Hp 2-2 and Hp 2-1 patients with diabetes had decreased CVD prevalence.

A possible relation between hs-CRP and Hp 2-2 has been examined in several studies. In patients with peripheral occlusive disease, Delanghe et al. [42] found a higher, yet statistically insignificant, hs-CRP in Hp 2-2 patients compared to Hp 1-1 and Hp 2-1 patient. Likewise, Braeckman et al. [43] found a higher, but also statistically insignificant, hs-CRP in apparently healthy Hp 2-2 individuals compared to Hp 1-1 and Hp 2-1 individuals. However, Delanghe et al. demonstrated in patients with essential hypertension higher hs-CRP in Hp 2-2 patients [44]. Recently, Mohieldein et al. found no association between Hp and markers of inflammation in diabetes [45]. Unfortunately, renal function was evaluated in none of these studies.

 The present study establishes a potential role for Hp 2-2 as a risk predictor of CVD in patients with CKD through an association with hs-CRP. Numerous studies have documented hs-CRP to be a valid and well-documented marker of inflammation [11] and hence a predictor of atherosclerosis and cardiovascular mortality in patients with IHD [13] as well as in patients with CKD, as recently demonstrated by Bazeley et al. [14].

 In patients with CKD, results on Hp genotype and CVD are conflicting. Pernod et al. found a prevalence of Hp 2-2 twice as high in end-stage renal disease patients with high CVD risk, defined as prior cardiovascular event, diabetes, or dyslipidemia, compared to end-stage renal disease patients with low CVD risk [46]. In a group of end-stage renal disease patients with diabetes, Burbea et al. [22] found a survival advantage in Hp 1-1 patients above 60 years, whereas patients below 60 years demonstrated a better survival when having Hp 2-2. However, Pernod et al. found no association between Hp genotype and cardiovascular mortality in patients with end-stage renal disease [23].

 Several mechanisms for the differences in antioxidant capability between Hp genotypes have been proposed. Lipid oxidation plays a crucial role in the initiation of atherosclerosis [47], and the first step in lipid oxidation is mediated by redox-active metals such as iron. Brouwers et al. demonstrated that Hp 2-2 is associated with high circulating levels of oxidised low-density lipoprotein thus posing a potential proatherogenic pathway for Hp 2-2 [48]. In an animal model of knock-in Hp 2-2 mice, Levy et al. demonstrated that Hp contributes to atherosclerotic plaque modulation by increasing the local inflammation. Hp 2-2 plaques accumulate more macrophages and have decreased levels of antiinflammatory cytokines [49]. Furthermore, Hp 2-2 has been associated with diminished nitric oxide bioavailability [50, 51], a crucial step in endothelial dysfunction and hence initiation of atherosclerosis. 

### 4.3. Limitations

Our study has some limitations. The cohort is relatively small and consists of patients with four stages of CKD. Furthermore, the underlying renal disease pattern is divers and comprises diseases with both high and low levels of inflammation. The design of the study is cross-sectional potentiating a risk of bias, and the causal inference cannot be established. 

## 5. Conclusion

In conclusion, in patients with CKD, a negative association was observed between Hp 2-2 and HRV. Furthermore, Hp 2-2 was positively associated with hs-CRP. Thus, Hp 2-2 may indicate a poor outcome in patients with CKD, and one may speculate that Hp 2-2 can help predict cardiovascular risk in patients with CKD as previously demonstrated in patients with diabetes. 

## Figures and Tables

**Table 1:** Clinical characteristics of patients with haptoglobin genotype 2-2 compared to patients with haptoglobin genotypes 1-1 and 2-1.

	Haptoglobin genotypes 1-1 and 2-1	Haptoglobin genotype 2-2	*P*
	(*n* = 40)	(*n* = 16)	
Age (y)	60 ± 11	56 ± 13	ns
Women (*n*)	18 (45%)	2 (13%)	*0.03 *
Smoking (*n*)	14 (35%)	4 (25%)	ns
Body mass index (kg/m²)	28 ± 5	30 ± 5	ns
Beta-blocker treatment (*n*)	16 (42%)	7 (44%)	ns
Haemodynamic parameters			
Systolic BP (mmHg)	142 ± 18	140 ± 18	ns
Diastolic BP (mmHg)	78 ± 10	80 ± 9	ns
Heart rate (beats per min)	72 ± 10	77 ± 11	ns
Laboratory parameters			
Creatinine clearance (mL/min)	35 ±18	45 ± 20	ns
Haemoglobin (mmol/L)	7.6 ± 1	8.0 ± 1	ns
Hs-CRP (mg/L)	2.5 (0.3; 24.0)	6.9 (0.6; 29.7)	*0.002 *
HRV indices			
RR (ms)	853 ± 124	790 ± 113	ns
SDNN (ms)	126 ± 37	100 ± 27	*0.02 *
SDNNindex (ms)	45 (39; 51)	39 (33; 45)	ns
SDANN (ms)	113 ± 36	90 ± 28	*0.04 *
pNN50 (%)	5.5 (3.5; 8.8)	3.4 (1.6; 7.1)	ns
RMSSD (ms)	24 (20; 30)	22 (17; 28)	ns

Values are mean ± SD or number of patients (%), except from high-sensitive C-reactive protein and heart rate variability indices SDNNindex, pNN50, and RMSSD which are given in geometric mean and ranges.

Abbreviations: BP: blood pressure, hs-CRP: high-sensitive C-reactive protein, and HRV: heart rate variability.

**Table 2:** Differences in SDNN, SDANN, and high-sensitive C-reactive protein between patients with haptoglobin genotype 2-2 compared to patients with haptoglobin genotypes 1-1 and 2-1.

	Difference between haptoglobin 2-2 and haptoglobins 1-1 and 2-1^†^ (95% CI)	*P* value
SDNN		
Crude*	−26.2 (−48; −4)	0.02
Adjusted**	−31.7 (−53; −10)	0.005
SDANN		
Crude*	−22.7 (−44; −1)	0.04
Adjusted**	−27.4 (−48; −7)	0.01
High-sensitive C-reactive protein		
Crude*	2.7 (2; 5)	0.002
Adjusted***	2.7 (1; 5)	0.003

^†^Difference presented as regression coefficient and 95%CI. Haptoglobin genotypes 1-1 and 2-1 are the reference level.

*Univariate linear regression analysis.

**Multivariate linear regression analysis adjusted for age, gender, heart rate, creatinine clearance, and beta-blocker treatment.

***Multivariate linear regression analysis adjusted for age, gender, body mass index, and smoking status.
